# Efficacy and safety of core stability training on gait of children with cerebral palsy: A protocol for a systematic review and meta-analysis

**DOI:** 10.1097/MD.0000000000018609

**Published:** 2020-01-10

**Authors:** Chuyao Huang, Yijun Chen, Guoming Chen, Yaying Xie, Jiahao Mo, Keyi Li, RuiLan Huang, Guanghua Pan, Yong Cai, Lei Zhou

**Affiliations:** a Guangzhou University of Chinese Medicine; b First Affiliated Hospital of Guangzhou Medical University; c National Clinical Research Center for Respiratory Disease; d First Affiliated Hospital of Guangzhou Medical University; e Third Affiliated Hospital of Guangzhou Medical University, Guangzhou, China.

**Keywords:** cerebral palsy, core stability, protocol, systematic review

## Abstract

**Background:**

Cerebral palsy (CP) is a common disability in children featured with pathological gait and limb function limitation due to muscle weakness. Improving limb function and quality of life is currently considered to be highlighted. Physiotherapy is a chief component of rehabilitation for children with CP, correcting gait and improve walking capacity through muscle strength training. Standard rehabilitation programs for CP have not been determined. Core stability training (CST), which coordinates limb balance via trunk control, is widely used in sports competition. And it is gradually introduced into the rehabilitation of children with cerebral palsy with a positive impact on the patients’ gait performance. By screening published literatures, this study aims to conduct a meta-analysis to systematically evaluate the effectiveness and safety of CST in gait of children with CP.

**Methods:**

Randomized controlled trials (RCTs) and controlled clinical trials (CCTs) on CST in the treatment of children with CP were searched from 6 databases. Moreover, the reference lists of conference papers and included literatures will be manually searched to avoid omissions. Literature screening and data extraction were performed independently by 2 researchers. RCTs carry out the risk of bias analysis evaluation from seven aspects through the Cochrane Collaboration's risk of bias tool. Fixed or random effect model will be performed to analyze the outcomes. When higher heterogeneity occurs (*I*^2^ > 50%), the sensitivity or subgroup analysis will also be conducted to find potential factors. And the Grading of Recommendations Assessment, Development and Evaluation (GRADE) approach is used for assessing the quality of evidence.

**Results:**

The study will evaluate the effect of CST on gait of children with CP from multiple outcomes, including walking speed, endurance, stride length, and safety.

**Conclusion:**

Based on evidence-based medicine, the conclusion of this study can demonstrate the effectiveness and safety of CST in gait correction for children with CP.

**PROSPERO registration number:**

PROSPERO CRD 42019134094.

## 1 Introduction

Cerebral palsy (CP) is a disease characterized by gait abnormalities because of myasthenia, spasticity, and impaired movement,^[1]^ which is the most common cause of physical impairment in children.^[2,3]^ According to some studies, about 2.1 of 1000 births will have the syndrome of cerebral palsy,^[4]^ while the prevalence in young people is as high as 74%.^[5]^ Children with CP have difficulties in activities, which severely affect the children's quality of life.^[6,7]^

There is no cure for cerebral palsy.^[8]^ WHO considers limb movement function as the main rehabilitation goal, and Keeratisiroj^[9]^ deems that the walking capacity training of CP needs to be taken seriously. The main intervention includes management of motor problems (physiotherapy, orthoses, medical treatment such as baclofen or diazepam),^[8]^ temporary medical interventions (baclofen and Botulinum toxin A injections), neurosurgical interventions, neuroprotection, orthopedic interventions.^[10]^ Due to the risks of neurosurgery and side effects of drugs intervention, physiotherapy becomes the core part of rehabilitation for children with CP.^[10,11]^ However, the effectiveness of physiotherapy remains uncertain because of the differences in rehabilitation programs, equipment, duration, and etc.^[12]^ Distal muscle strength training is generally the main component of rehabilitation. Actually, core exercises also play an important role in the movement and balance of distal limb strength.^[13]^

Core stability training (CST) is a popular strength training in sports at first and gradually applied to rehabilitation medicine. It refers to the ability to control the muscle tissue around the lumbar pelvic cavity in order to stabilize the spine and transfer power from the trunk to the limbs.^[14–16]^ CST also has been confirmed that has a positive influence on dynamic sitting and standing balance, trunk control, and gait.^[17]^ A study has found that adding CST to the rehabilitation program can significantly improve the endurance time of trunk muscles and gait in children with hemiplegic CP.^[18]^ Since children with CP commonly have a syndrome of myasthenia in the trunk, CST will be a feasible intervention to alleviate the pain of patients and improve the quality of patients’ life.

Although there are several studies for CST on gait of children with cerebral palsy, no systematic and meta-analysis concerning CST on gait of children with cerebral palsy are found. Therefore, the aim of this systematic review and meta-analysis is to access the efficacy and safety of CST on gait of children with cerebral palsy according to the current studies.

## 2 Methods

### 2.1 Ethics and dissemination

Since this is a systematic review and there is no privacy data, ethical approval and informed consent are not necessary. The results of this article can be disseminated as much as possible, such as in peer-reviewed publications and conference presentations.

### 2.2 Inclusion criteria for study selection

#### 2.2.1 Types of studies

All the RCTs and CCTs that applying the CST on gait of children as the treatment for CP will be brought into the review, excluding other types of studies which include data error and incomplete clinical trials. Blind method, allocation concealment and randomization method are not restricted. Moreover, no language or publication status limitation will be eliminated.

#### 2.2.2 Types of participants

Children diagnosed with CP aged 7 to 12 years are characterized by gait abnormalities and there are no limitations on sex and race. Besides, the medical diseases combined will be eliminated and the studies that do not meet diagnostic criteria will be ruled out. The length of illness and the severity of illness should be taken into account.

#### 2.2.3 Types of interventions

The main interventions of the treatment group includes CST, and other clinical treatments and rehabilitation training were in accordance with the control group, which adopts conventional rehabilitation treatment for CP, such as exercise therapy, physical therapy, occupational therapy, botulinum toxin injections, and etc.

#### 2.2.4 Types of outcome measures

##### 2.2.4.1 Primary outcomes

1. Walking speed
2. Stride length
3. Step length

##### 2.2.4.2 Secondary outcomes

1. Endurance
2. Kinematic parameters
3. Safety

### 2.3 Search methods for the identification of studies

#### 2.3.1 Electronic searches

A systematic electronic search was performed by 2 researchers through 6 databases from their inception to the present date: PubMed, Embase, Web of Science, Cochrane, EBSCO, OVID. We will build search strategy adopting a combination of keyword and free word: keyword is “cerebral palsy”, “core stability”, “gait”, “children” and “randomized controlled trials”. Detailed search strategy will be shown in Table 1.

**Table 1:**
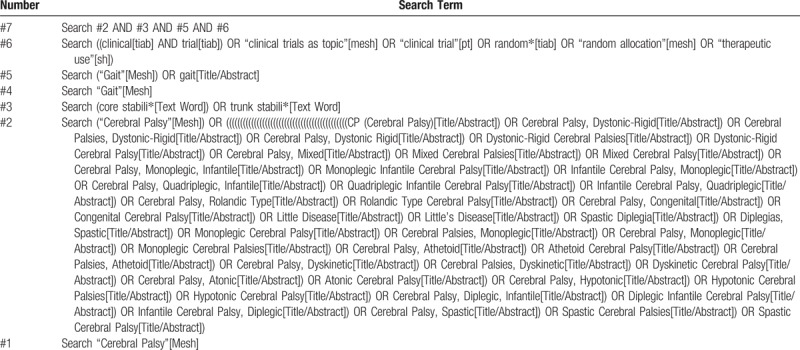
Search strategy for the PubMed database.

#### 2.3.2 Other searches

Considering the possible omissions, we retrieve the conference papers. Moreover, the reference lists of included studies are checked for potential studies.

### 2.4 Selection of studies

**Figure 1:**
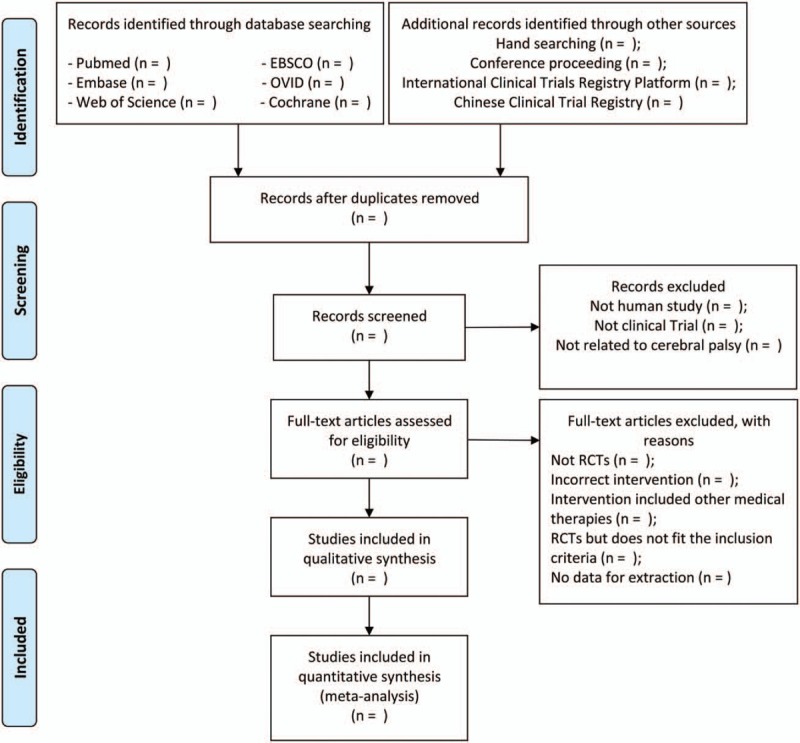
Flow diagram of study selection process.

Study selection will be cross-checked with 2 independent studies (YX and JM). Document management EndnoteX9 will be used the collected documents to import and duplicate documents will be deleted. First, the investigators will rule out obvious nonconformities by title and abstract. Then the investigators will carefully examine the full text according to the inclusion and exclusion criteria established earlier. The entire filtering process will be shown in a flowchart (Fig. 1).

### 2.5 Data extraction and analysis

#### 2.5.1 Data extraction and management

The data are extracted by 2 researchers using a predetermined form of data collection. The extracted data should include but not be limited to the following items: title, first author, publication time, sample size, severity, duration, results, and adverse events. For the inconsistencies, the two researchers will discuss and negotiate with a third researcher.

#### 2.5.2 Coping with the questionable data

In the event of data inconsistencies, errors or omissions, the investigators will contact the first author to confirm whether the data is correct or missing. In addition, the potential impact of suspect data will be discussed in the discussion section.

#### 2.5.3 Quality of evidence

To appraise the evidence quality more objectively, the review will use the Grading of Recommendations Assessment, Development and Evaluation (GRADE) and complete the Summary of Findings table.

#### 2.5.4 Assessment of risk of bias in included studies

The risk of bias is assessed by the 2 reviewers using the Cochrane Handbook for Systematic Reviews of Intervention. The researchers will use “L”, “U” and “H” for low risk, uncertainty, and high risk, respectively. In the appraisal, 7 sectors will be assessed, including random sequence generation, allocation concealment, blinding of participants and personnel, blinding of outcome assessment, completeness of outcome data, selective reporting, and other sources of bias. Potential disagreements will be discussed with a third reviewer, and corresponding authors will be contacted as needed.

### 2.6 Data analysis

#### 2.6.1 Data synthesis

The system review will be done by using RevMan 5.3. MD, RR with fixed or random effect models will be used for calculation. To appraise the treatment effect for continuous data, mean difference (MD) in terms of 95% confidence interval (CI) will be applied, just as relative risk (RR) for dichotomous data. In addition, if heterogeneity is considered significant, sensitivity or subgroup analysis will be generated to distinguish its sources. When there is insufficient data for quantitative analysis, the review will only represent and summarize the evidence.

#### 2.6.2 Assessment of heterogeneity

On the basis of data analysis, the random or fixed effect model will be determined according to the statistical value of heterogeneity *I*^*2*^. Specifically, those with high heterogeneity (*I*^*2*^ < 50%) will adopt the fixed-effect model, and if the results are contrary, the random-effect model will be applied.

#### 2.6.3 Sensitivity analysis

As mentioned earlier, sensitivity analysis is completed when heterogeneity is greater than 50%. Specifically, the meta-analysis will be reconfirmed whether the low-quality studies, small samples or older people influence the results.

#### 2.6.4 Subgroup analysis

The subgroup analysis will observe substantial heterogeneity and find out the reasons. Outcome types, duration of disease, quality of the study, patient ethnicity and so on will be included in the subgroup analysis.

#### 2.6.5 Assessment of reporting bias

When the research literature is sufficient with at least 10 RCTs or CCTs, the reported bias will be visualized by the funnel plot. If the funnel chart is asymmetric, Begg and Egger test will be completed. The value of *P* > .05 will be interpreted as reporting bias without significance. Since the asymmetry of funnel plots does not represent the actual bias of data, the following terms are used to explain potential possibilities, such as low methodological quality, small data sample size, or true heterogeneity.

## 3 Discussion

Cerebral palsy is a non-progressive neurological disorder, manifested with mobility dysfunction and other additional impairment (cognitive difficulties, sensibility and senses impairment, etc).^[19–21]^ It is a permanent disorder with no cure currently and therefore its managements are usually intent on how to improve patients’ quality of life and prevent exacerbation.^[8]^ Given that the prominent complaint of CP is mobility impairment, promising interventions to help improve motor disability have long been the focus on the treatment of children with CP.^[22–24]^

CST, a training of lumbar-pelvic-hip complex, is used not only in exercises and sports training, but also as a popular kind of physiotherapy in rehabilitation of motor disability. Studies have also reported that CST could improve trunk function, balance and motor proficiency, in which the constant cooperation of the passive (vertebrae, intervertebral discs, ligaments, etc), active (the core musculature) and neural control subsystems may play a key role.^[15,18,25–29]^ However, most relevant studies are small series and offer limited evidence to the efficacy and safety for CST on gait of children with CP. Consequently, this study is the first to provide a systematic review and meta-analysis of the efficacy and safety for CST on gait of children with CP. We hope that this study can help provide a further insight on the management of CP. Nevertheless, it may not provide the highest level of evidence in that:

(1) the patient-specific ways of CST in different trials may cause the heterogeneity;
(2) the language limitation (English only) during searching may bring some risks of bias.

## Author contributions

**Data curation:** Yijun Chen, Yaying Xie, Jiahao Mo, Keyi Li, RuiLan Huang, Yong Cai, Lei Zhou.

**Methodology:** Chuyao Huang, Yijun Chen, Guoming Chen, Yaying Xie, Jiahao Mo, Keyi Li.

**Writing – original draft:** Chuyao Huang, Yijun Chen, Guoming Chen, Yaying Xie, Jiahao Mo, Keyi Li, RuiLan Huang.

**Writing – review & editing:** Chuyao Huang, Yong Cai, Lei Zhou.

Guanghua Pan orcid: 0000-0001-9521-1932.
